# Integrating Experimental Measurements, WinXCom Calculations and Monte Carlo Simulations to Evaluate the Ionizing Radiation Shielding Performance of UPR/Bi_2_WO_6_ Nanocomposites

**DOI:** 10.3390/polym18161955

**Published:** 2026-08-10

**Authors:** İsa Emin Ongun, Yaşar Karabul

**Affiliations:** 1 Graduate School of Natural and Applied Sciences, Yildiz Technical University, İstanbul 34220, Türkiye; 2 Department of Physics, Faculty of Arts and Sciences, Yildiz Technical University, İstanbul 34220, Türkiye

**Keywords:** Bi_2_WO_6_ nanoparticles, unsaturated polyester resin, gamma-ray shielding, polymer nanocomposites, MONTE Carlo simulation

## Abstract

Lead-free polymer nanocomposites have attracted considerable attention as sustainable alternatives for ionizing radiation shielding. In this study, unsaturated polyester resin (UPR) nanocomposites containing hydrothermally synthesized Bi_2_WO_6_ nanoparticles (2.5–10 wt.%) were fabricated, and their gamma-ray shielding performance was evaluated experimentally and theoretically. The structural and morphological properties of the nanoparticles were characterized by XRD, FTIR, and FESEM. Mass attenuation coefficient (MAC), half-value layer (HVL), and mean free path (MFP) were experimentally determined at photon energies of 81–1332 keV and validated using WinXCom calculations and MCNP6.3 Monte Carlo simulations. In addition, the effective atomic number (Z*_eff_*) and effective electron density (N*_eff_*) were calculated over the energy range of 0.001–100 MeV. Increasing the Bi_2_WO_6_ content enhanced the MAC, Z*_eff_*, and N*_eff_* values while reducing the HVL and MFP, indicating improved shielding efficiency. The composite containing 10 wt.% Bi_2_WO_6_ exhibited the highest attenuation performance, achieving an MAC of 0.4638 cm^2^ g^−1^ at 81 keV. The maximum deviation between experimental, theoretical, and simulation results remained below 2.8%, demonstrating excellent agreement. These findings identify Bi_2_WO_6_-reinforced UPR nanocomposites as promising lightweight, lead-free materials for gamma-ray shielding applications.

## 1. Introduction

Recent technological advances have significantly increased the use of ionizing radiation across a wide range of fields, from healthcare and nuclear physics to scientific research, industrial applications, food sterilization, geological research, and military/security applications [1,2]. Ionizing radiation, or high-energy photon radiation, can cause serious damage to biological tissues and structural materials due to its high penetration capability. Therefore, the development of radiation shielding materials that can protect both living organisms and structural components to minimize the harmful effects of radiation has become a critical necessity [3,4]. Although lead is one of the most effective conventional radiation shielding materials due to its high atomic number and density, its toxicity, weight due to its high density, limited flexibility, and low mechanical strength are among the significant disadvantages that restrict its use [2,5]. Therefore, researchers have long been motivated to develop new non-toxic, cost-effective, and lightweight shielding materials [4,6]. Recent studies on polymer–nanoparticle, mineral, and metal-based composite systems show that their mechanical, thermal, and functional properties can be improved by appropriately designing the composition and microstructure of these structures [7]. Furthermore, integrating high-atomic-number filler materials into the composite structure contributes to improving radiation attenuation performance by increasing the probability of interaction with photons [8,9]. Based on this understanding, in recent years, various high-atomic-number metals or compounds containing these metals, including Ba, Bi, W, Gd, Zr, Fe, Ni, etc., have been used as micro- or nano-sized additives to polymers for the purpose of attenuating ionizing radiation [10,11]. While polymer matrices are preferred due to their low density, easy processability, low cost, and good mechanical properties, metal and metal oxide nanoparticles containing high Z elements significantly improve the radiation attenuation performance of composites by increasing the probability of interaction with photons. In this context, numerous radiation shielding composites have been developed using heavy metal oxides such as Bi_2_O_3_, WO_3_, BaWO_4_, Gd_2_O_3_ and Bi_2_WO_6_ in different polymer matrices such as PVC, PMMA, PTFE, epoxy and PVP/PEG [4,12,13,14,15,16,17,18,19].

Existing studies show that the atomic number, density, concentration and distribution of the filler material in the composite are the fundamental parameters determining the radiation attenuation performance [20]. In general, it has been reported that increasing the amount of high Z additive increases the linear attenuation coefficient and mass attenuation coefficient values; however, parameters such as half-value layer (HVL), one-tenth-value layer (TVL) and mean free path (MFP) decrease. In addition, it has been shown that hybrid filler systems and different metal oxide combinations can provide improved shielding performance compared to single-component composites [21,22].

Among high atomic number dopants, Bi_2_WO_6_ stands out due to its combination of both bismuth (Z = 83) and tungsten (Z = 74) in its structure. Rabiei and colleagues compared PVC composites prepared with WO_3_, BaWO_4_, and Bi_2_WO_6_ nanoparticles; they reported that Bi_2_WO_6_-doped composites exhibited the highest linear and bulk attenuation coefficients over a wide X-ray energy range, while showing the lowest HVL and TVL values. They attributed this superior performance to Bi_2_WO_6_’s ability to contain two high atomic number elements, bismuth and tungsten, in the same crystal structure, resulting in a denser, less porous composite structure [23]. In addition, it was shown that lightweight and lead-free radiation shielding materials can be produced by developing Bi_2_WO_6_-based nanofiber membranes and flexible hybrid structures [24]. In composite materials, not only the chemical structure of the filler but also the particle size and microstructural distribution significantly affect the radiation shielding performance. Nanometer-sized fillers can increase the probability of interaction with photons thanks to their higher specific surface areas and more homogeneous distribution within the polymer matrix [1]. Therefore, many studies have reported that nanoparticle-reinforced composites show higher radiation attenuation performance compared to composites containing micrometer-sized fillers with the same chemical composition. For example, several studies comparing micro- and nano-sized Bi_2_O_3_ or WO_3_ fillers within the same polymer matrix have demonstrated that nano-WO_3_ or nano Bi_2_O_3_-reinforced composites exhibit higher photon attenuation coefficients and lower HVL values than their micro-counterparts, primarily owing to the more homogeneous dispersion and larger interfacial area of nanoparticles [25,26,27].

The vast majority of studies reported in the literature have focused on PVC, PMMA, PTFE, and epoxy-based composite systems [4,28]. Despite the growing interest in UPR-based radiation shielding composites, most reported studies have focused on conventional fillers such as Bi_2_O_3_, SnO_2_ and Bi_2_(WO_4_)_3_ [29,30,31], whereas the radiation shielding characteristics of Bi_2_WO_6_ nanoparticle-reinforced UPR nanocomposites have not yet been systematically investigated. Additionally, UPR is particularly attractive as a matrix for rigid radiation shielding applications because of its low cost, ease of processing, dimensional stability, chemical resistance, and the well-established mechanical performance of the commercial resin. These advantages make UPR an excellent matrix for developing lightweight, lead-free functional radiation shielding composites.

Motivated by the above findings reported in previous studies and the attractive characteristics of UPR as a polymer matrix, UPR nanocomposites reinforced with Bi_2_WO_6_ nanoparticles at different weight fractions were fabricated in this study to investigate their ionizing radiation shielding performance. The structural and morphological properties of the components of the composites were characterized in detail. The radiation attenuation performance of the prepared composites against ionizing radiation was evaluated using experimental measurements, WinXCom theoretical calculations, and MCNP Monte Carlo simulations. In this context, basic radiation shielding parameters such as linear and mass attenuation coefficients, HVL, MFP, effective atomic number (Z*_eff_*), and electron density (N*_eff_*) were determined, and the experimental results were validated by comparing them with theoretical and simulation data. Thus, the effect of Bi_2_WO_6_ nanoparticles on the shielding performance of UPR-based composites against ionizing radiation was revealed in detail, and a comprehensive evaluation was presented for the development of lead-free, high-performance polymer-based radiation shielding materials.

## 2. Materials, Methods and Structural Characterizations

### 2.1. Materials

Bismuth nitrate pentahydrate (Bi(NO_3_)_3_·5H_2_O, CAS No: 10035-06-0) and polyvinylpyrrolidone (PVP, CAS No: 9003-39-8), used as the precursor materials for Bi_2_WO_6_ synthesis, were purchased from Sigma-Aldrich (Toluca, Mexico and St. Louis, MO, USA, respectively). Sodium tungstate dihydrate (Na_2_WO_4_·2H_2_O, CAS No: 10213-10-2) was also procured from Sigma-Aldrich (New Delhi, India). All chemicals were of analytical grade and used directly as received without any further purification.

An orthophthalic unsaturated polyester resin (Polipol 3455, Poliya Composite Resins and Polymers, Istanbul, Türkiye) featuring low reactivity and medium viscosity was employed as the polymer matrix. This general-purpose, casting-type resin is characterized by a density of 1.12–1.13 g/cm^3^, a viscosity range of 500–800 cP, a monomer content of 36–39%, and a typical gel time of 6–9 min, making it highly suitable for general composite manufacturing applications. According to the manufacturer’s technical datasheet, the neat cast and post-cured resin exhibit a tensile strength of 66 MPa, flexural strength of 138 MPa, tensile modulus of 3047 MPa, and Izod impact strength of 11 kJm^−2^. These manufacturer-reported properties were considered sufficient to justify the selection of UPR as the matrix material for rigid radiation shielding applications. Since the primary objective of the present study was to evaluate the radiation attenuation performance of the developed nanocomposites, additional mechanical characterization of the fabricated bulk composites was considered beyond the scope of this work.

To initiate polymerization at room temperature, a dedicated curing system consisting of methyl ethyl ketone peroxide (MEK-P) (Butanox M-60, containing 9.9% active oxygen) as the catalyst and cobalt 2-ethylhexanoate (containing 6 wt.% Co) as the accelerator was utilized. The resin, along with its corresponding hardener and accelerator components, was supplied by Poliya Composite Resins and Polymers, and all materials were used as received without further purification.

### 2.2. Synthesis of Bi_2_WO_6_ and the Preparation Procedure of the UPR Based Nanocomposites

Bi_2_WO_6_ nanoparticles were synthesized via a hydrothermal method adapted from the procedure reported by Derikvand and Tahmasebi [32]. In the initial stage, 1.11 g of Na_2_WO_4_·2H_2_O was dissolved in 50 mL of deionized water and stirred magnetically for 1 h at room temperature to form a tungstate solution. Concurrently, a separate mixture was prepared by dissolving 2.13 g of Bi(NO_3_)_3_·5H_2_O, 0.50 g of PVP, and 0.60 mL of HCl in 50 mL of deionized water under continuous stirring for 30 min. The tungstate solution was then introduced dropwise into the bismuth-containing solution. After further stirring for 10 min, the obtained suspension was transferred into a 200 mL Teflon-lined stainless steel autoclave. The autoclave was maintained in an oven at 180 °C for 12 h and subsequently allowed to cool naturally to room temperature. The as-prepared precipitate was isolated by centrifugation, followed by multiple washing cycles with deionized water and ethanol. Finally, the collected powder was dried at 80 °C for 4 h. Following its structural characterization (see Section 2.3), the synthesized Bi_2_WO_6_ powder was utilized as a reinforcing agent in UPR-based composites.

**Figure 1:**
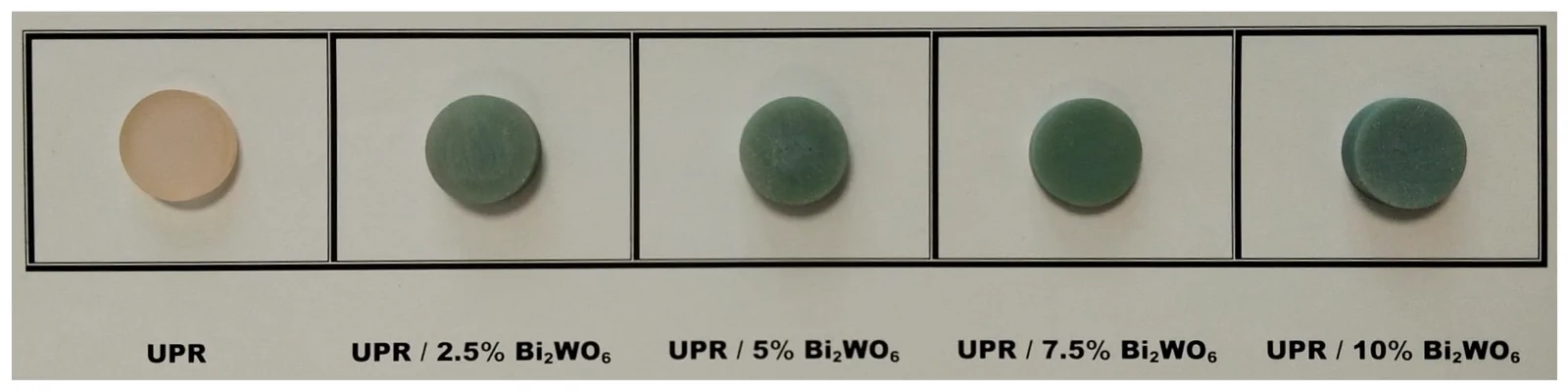
The photographs of the pure UPR and the UPR/Bi_2_WO_6_ nanocomposites.

Following the synthesis of Bi_2_WO_6_, neat UPR specimens were prepared according to the procedure illustrated in Figure 1. The process initiated with the allocation of an appropriate amount of UPR into a beaker. To initiate curing, 0.3 wt.% cobalt octoate (CoO) and 3 wt.% methyl ethyl ketone peroxide (MEK-P) were incorporated into the resin matrix as an accelerator and a catalyst, respectively. The resulting mixture was gently agitated to minimize air bubble entrapment while ensuring a completely homogeneous blend. Immediately after thorough homogenization, the resin was cast into cylindrical Teflon molds that had been pre-treated with a mold release agent. Finally, the neat UPR samples were allowed to cure at room temperature for 24 h before being demolded.

For the preparation of UPR/Bi_2_WO_6_ nanocomposites, a similar procedure was followed with the integration of a particle dispersion stage. Initially, the synthesized Bi_2_WO_6_ nanoparticles were added to the unsaturated polyester resin at predetermined weight fractions. To ensure uniform dispersion and minimize nanoparticle agglomeration, the mixture was subjected to ultrasonic processing in a sonicator bath for 30 min, followed by high-speed magnetic stirring at 500 rpm for 30 min. Once a homogeneous dispersion was achieved, 0.3 wt.% cobalt octoate (CoO) and 3 wt.% methyl ethyl ketone peroxide (MEK-P) were introduced into the suspension. The mixture was gently agitated to prevent air bubble entrapment. Immediately after homogenization, the mixture was cast into the same cylindrical Teflon molds pre-treated with a mold release agent. Finally, the resulting nanocomposite specimens were allowed to cure at room temperature for 24 h prior to demolding. The cured neat UPR and UPR/Bi_2_WO_6_ nanocomposite specimens were then demolded and prepared for subsequent characterization, as shown in Figure 1. It should also be noted that the maximum Bi_2_WO_6_ loading investigated in the present study was limited to 10 wt. Preliminary experiments were also carried out using Bi_2_WO_6_ loadings higher than 10 wt.%. Although theoretical WinXCom calculations predicted a continued increase in the attenuation parameters with increasing filler content, the agreement between the experimental measurements and both the WinXCom (Version 1.0) calculations and MCNP simulations deteriorated at these higher loadings. This behavior is attributed to the difficulty of achieving homogeneous nanoparticle dispersion as the resin viscosity increased significantly during processing. The resulting particle agglomeration and local compositional inhomogeneities cause the fabricated composites to deviate from the homogeneous material assumption employed in the theoretical and Monte Carlo models. For this reason, the present study focused on compositions up to 10 wt.% Bi_2_WO_6_, which provided the best overall agreement between experimental, theoretical, and simulation results while maintaining satisfactory processability and composite homogeneity.

To mitigate experimental uncertainties arising from specimen defects, detector variations, and material inhomogeneities, each composite sample was fabricated with a uniform diameter of 10 mm at two distinct thicknesses. After curing at room temperature for 24 h, the specimens were successfully demolded. To ensure dimensional accuracy, the final thicknesses of the prepared samples were verified using a Mitutoyo micrometer (Mitutoyo Corp., Kawasaki, Japan), and the measured values are presented below:

UPR: 9.85 mm/18.20 mm; UPR/2.5% Bi_2_WO_6_: 10.15 mm/19.12 mm; UPR/5% Bi_2_WO_6_: 11.28 mm/18.84 mm; UPR/7.5% Bi_2_WO_6_: 10.71 mm/19.64 mm; UPR/10% Bi_2_WO_6_: 10.37 mm/18.93 mm.

The mass densities of the samples (ρ), an important factor in radiation shielding performance, were determined experimentally according to Archimedes’ principle. The relevant experiment was carried out by measuring the masses of the samples in air and water environments; the bulk densities of the samples were obtained as shown in Table 1. The relative heaviness (RH) relative to lead (Pb) is also given in Table 1, as it allows the densities of the developed composites to be expressed as a percentage relative to lead density. The RH value was calculated using $R_{H}=\left(\frac{\rho_{sample}}{\rho_{Pb}}\right)\times 100$ relation.

### 2.3. Physical and Chemical Characterizations

In this section, the structural characterization of the synthesized Bi_2_WO_6_ was first carried out to evaluate its morphology, elemental composition, and crystal structure using FESEM, SEM-EDS, and XRD. FESEM imaging and SEM-EDS analysis were performed on a Thermo Fisher Apreo 2S scanning electron microscope, while the XRD pattern was recorded using an XPERT PRO X-ray diffractometer. Subsequently, the chemical properties of the composite constituents (Bi_2_WO_6_) and the fabricated nanocomposites were investigated via FTIR spectroscopy (Bruker Tensor 27) to confirm the formation of the composite structure.

**Figure 2:**
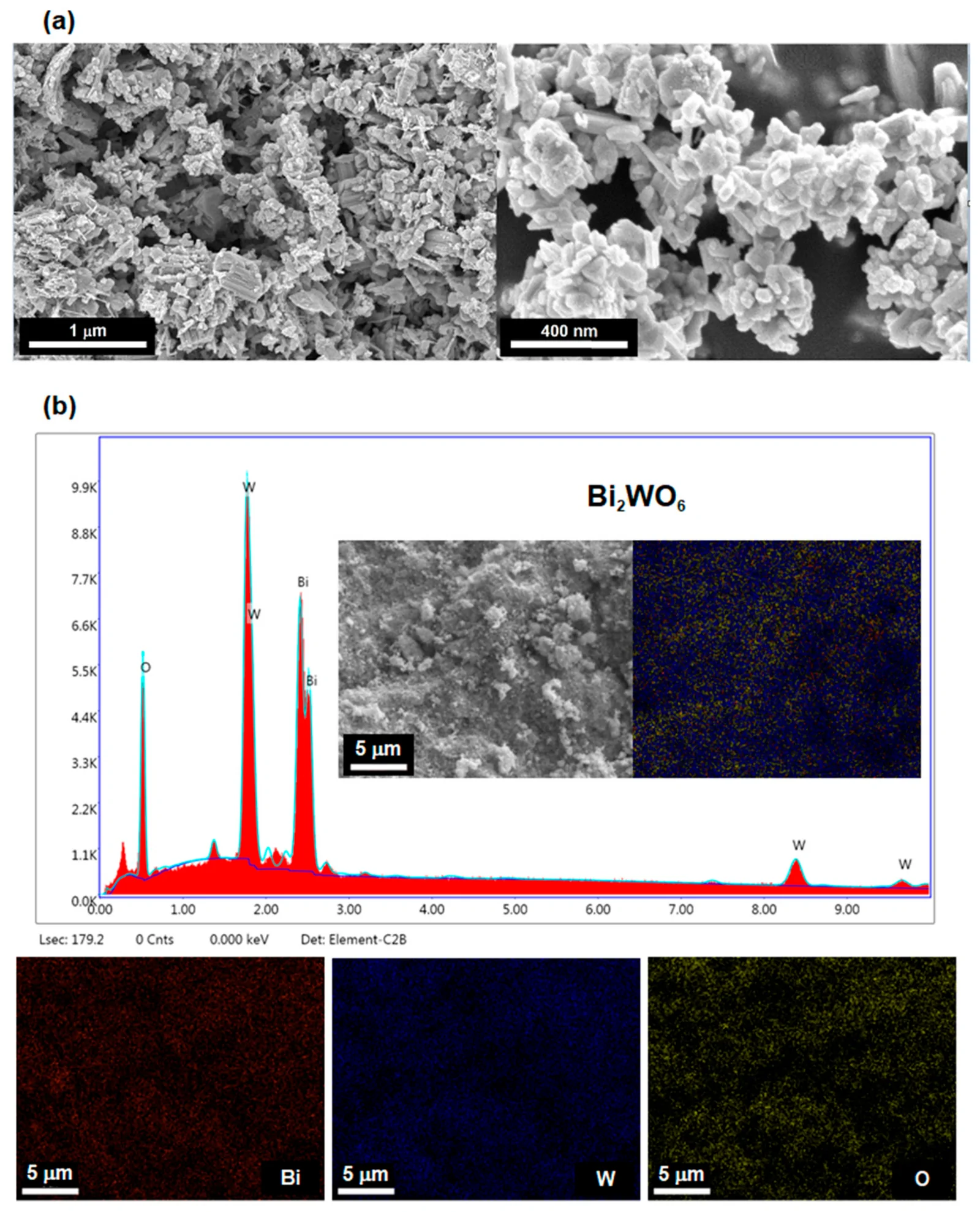
(**a**) FE-SEM images of the synthesized Bi_2_WO_6_ nanoparticles at different magnifications (1 µm scale (**left**) and 400 nm scale (**right**) and, (**b**) EDS elemental mapping and spectrum. In the elemental maps, Bi, W, and O are represented in orange, blue, and yellow, respectively.

Figure 2a shows FESEM images of the Bi_2_WO_6_ sample obtained at different magnifications. In the low magnification image (1 μm scale), the sample is seen to have a three-dimensional clustered and porous morphology. At 1 μm magnification, these clusters are thought to be formed by the aggregation of irregularly oriented plate-like and/or flaky crystals. However, as can be seen from the higher magnification image (400 nm scale), Bi_2_WO_6_ is in the form of flower-like microstructures consisting of thin sheet-like subunits. Based on approximate assessments from FESEM images, the dimensions of the plate-like subunits range from 50–150 nm, while the diameters of the secondary floral structures formed by these subunits range from approximately 300–800 nm. The stacking and radial growth of the nanosheets forming the structure have resulted in a porous morphology with a high surface area.

**Figure 3:**
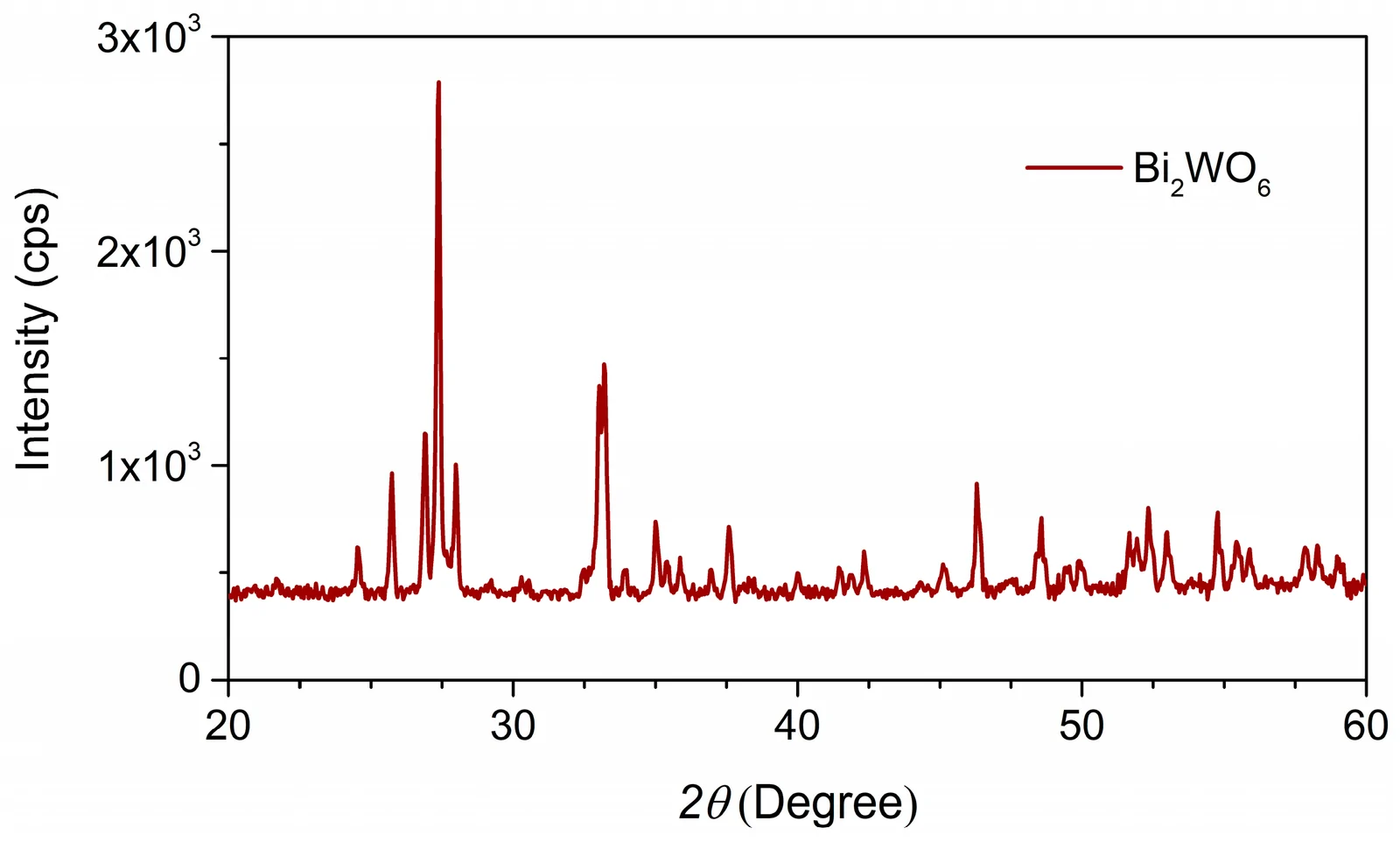
The XRD pattern of synthesized Bi_2_WO_6_ nanoparticles.

According to the EDX spectrum and elemental distribution maps of the Bi_2_WO_6_ sample given in Figure 2b, characteristic peaks of Bi, W, and O elements are clearly observed in the sample. The absence of any distinct peak for foreign elements in the spectrum indicates that the sample was synthesized with high purity. This also supports the formation of single-phase Bi_2_WO_6_, consistent with the XRD results given in Figure 3. The elemental mapping results reveal that Bi, W, and O elements are homogeneously distributed on the sample surface.

The phase purity and crystal structure of the hydrothermally synthesized Bi_2_WO_6_ nanoparticles were investigated by X-ray diffraction (XRD). As shown in Figure 3, all the observed diffraction peaks are sharp and well-defined, indicating a high degree of crystallinity. The characteristic diffraction peaks located at *2θ* values of approximately 28.3°, 32.8°, 47.1°, 55.8°, and 58.5° can be explicitly indexed to the (131), (200), (202), (133), and (262) crystal planes, respectively. These results confirm that the sample crystallizes in the well-crystallized orthorhombic phase of Bi_2_WO_6_ (russellite), which perfectly matches the standard JCPDS Card No. 39-0256. No trace of impurity peaks (such as Bi_2_O_3_ or WO_3_) was detected, implying the high chemical purity of the synthesized product. This XRD pattern is in excellent agreement with the diffraction profile reported by Derikvand and Tahmasebi [31], whose hydrothermal synthesis protocol was adapted in this study.

The average crystallite size (D) of Bi_2_WO_6_ nanoparticles was estimated using the Debye–Scherrer equation:

(1) $$D=\frac{K\lambda }{\beta cos\theta }$$

In Equation (1), K is the shape factor (0.9), λ is the X-ray wavelength, β is the full width at half maximum of the diffraction peak (FWHM), and θ is the Bragg diffraction angle. The most intense diffraction peak found at 27.38° was used for the calculation of D. The D was estimated to be approximately 49.9 nm, representing the nano crystalline structure of the synthesized Bi_2_WO_6_. It is useful to note at this stage that the D obtained from the Debye–Scherrer equation represents the size of the coherently diffraction areas and may differ from the particle size observed in the FE-SEM images.

**Figure 4:**
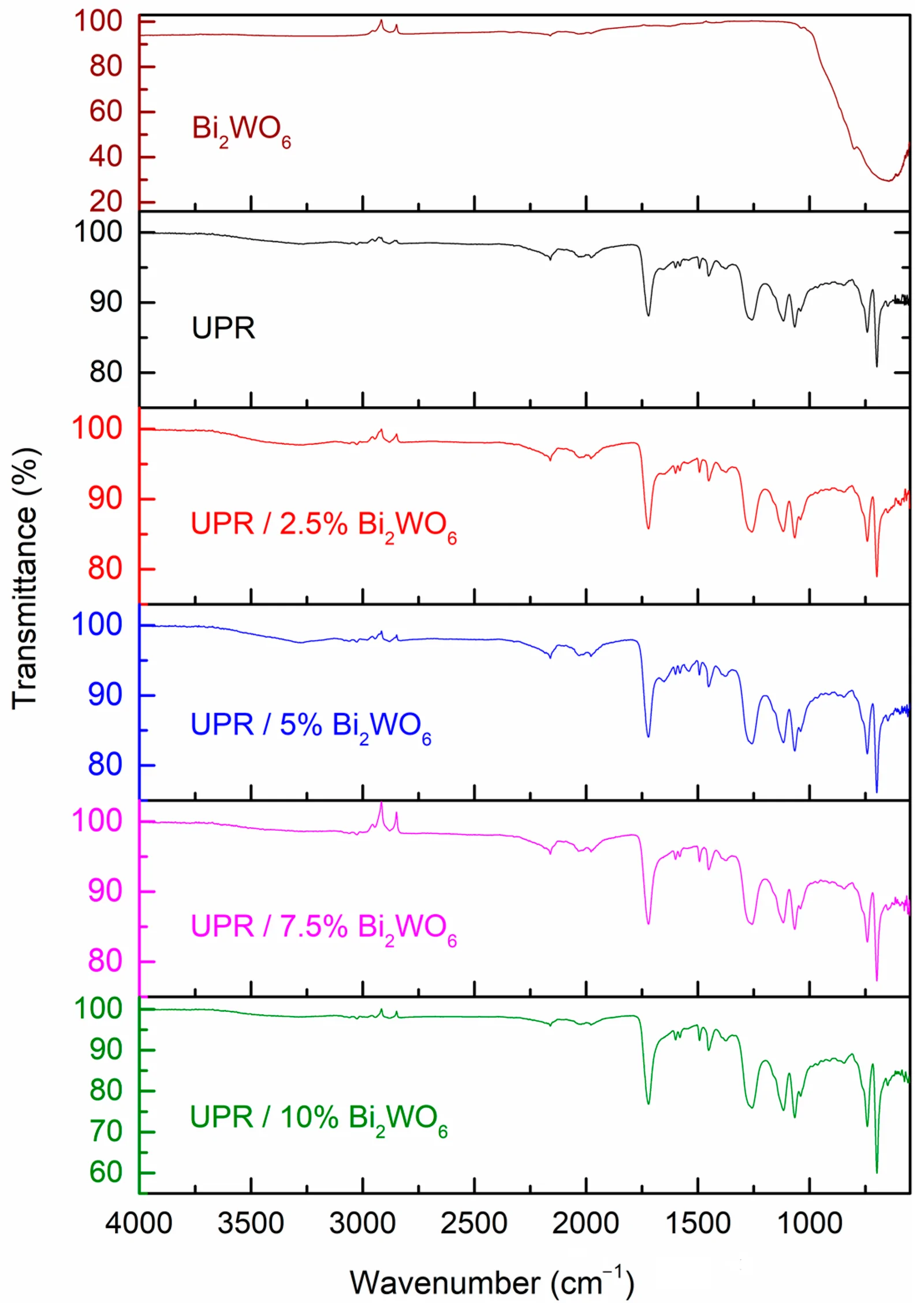
FTIR spectra of the neat UPR, reinforcing nanoparticle Bi_2_WO_6_, and the UPR/Bi_2_WO_6_ nanocomposites.

The FTIR spectra of UPR, the main component of the composites, Bi_2_WO_6_, the additive, and the UPR/Bi_2_WO_6_ nanocomposites are given in Figure 4 in the wavenumber range of 4000–550 cm^−1^. The broad band observed around 3600 cm^−1^ in the spectrum of the pure UPR sample (see Figure 4) is attributed to hydroxyl (O–H) groups in the polyester chain. Bands observed in the lower wavenumber range of 3000–2700 cm^−1^ correspond to aliphatic C–H stretching vibrations. Additionally, the band observed at approximately 1719 cm^−1^ represents carbonyl (C=O) stretching vibrations associated with ester groups. The band detected around 1615 cm^−1^ belongs to C=C vibrations of the aromatic ring. Bands observed at 1260 and 1124 cm^−1^ correspond to asymmetric and symmetric C–O–C stretching vibrations in the aromatic ester structure, respectively. The simultaneous observation of all these characteristic bands confirms the chemical structure of the UPR matrix, the main component of the composites [33]. On the other hand, the broad absorption band observed in the 600–1000 cm^−1^ region in the Bi_2_WO_6_ sample originates from the stretching vibrations of W–O and Bi–O bonds and the vibrational modes of W–O–W bridging bonds (See Figure 4) [34].

Comparing the FTIR spectra of pure UPR and Bi_2_WO_6_-doped UPR nanocomposites reveals that the characteristic bands of the polyester matrix are preserved in all samples. This indicates that the addition of Bi_2_WO_6_ does not alter the fundamental chemical structure of UPR. In contrast, significant changes in band intensities and peak shapes are observed, particularly in the fingerprint region between 650–550 cm^−1^, depending on the doping ratio. These changes are due to the overlap of Bi–O, W–O, and W–O–W lattice vibrations of the Bi_2_WO_6_ crystal structure with the existing vibrational bands of UPR. Furthermore, the absence of new absorption bands or significant shifts in the characteristic UPR bands suggests that the nanocomposites are formed through physical mixing rather than chemical reaction. Although FTIR does not provide a quantitative assessment of nanoparticle dispersion, the progressive evolution of the characteristic Bi_2_WO_6_ bands with increasing filler content supports the successful incorporation of Bi_2_WO_6_ nanoparticles into the UPR matrix and is consistent with a reasonably homogeneous distribution within the investigated composition range. This interpretation is further supported by the close agreement between the experimental attenuation measurements, WinXCom calculations, and MCNP simulations for composites containing up to 10 wt.% Bi_2_WO_6_.

### 2.4. Radiation Shielding Parameters

In radiation shielding studies, transmission factors (TFs) are commonly evaluated to represent the fraction of radiation penetrating a given shielding medium. The calculation of TFs is fundamentally based on Beer–Lambert’s law, which describes the exponential attenuation in beam intensity resulting from absorption and scattering processes inside the shielding material. This relationship is formally expressed through the equation shown in Equation (2).

(2) $$I=I_{0}e^{-\mu x}$$

Here, I_0_ is the radiation intensity before interaction with the shielding medium, I is the intensity of the radiation transmitted after passing through the radiation material, μ is the linear attenuation coefficient (or LAC), and x is the thickness of the shielding material in centimeters. LAC, determined in the study included in Equation (2) and considered as the first TF parameter, expresses how effectively the incident photon is attenuated per centimeter in the material. This attenuation is influenced by both the radiation energy and the elemental structure of the shielding structure. When LAC is normalized according to material density, the mass attenuation coefficient (MAC or μ/ρ), usually given in cm^2^/g, is obtained. Another TF parameter presented in Equation (3), the half-value thickness (HVL), represents the thickness of the radiation absorber material required to reduce the incident photon intensity to 50% of its original value and is considered a practical measure of radiation shielding efficiency.

(3) $$HVL=\frac{0.693}{\mu }$$

The tenth value layer (TVL) also refers to the thickness of radiation shielding material required to reduce the incident radiation intensity to 10% as specified in Equation (4),

(4) $$TVL=\frac{2.302}{\mu }$$

Mean free path (MFP) describes the average distance travelled by the incident photon before the next interaction within the material. MFP, calculated by Equation (5), is another critical parameter in evaluating the decrease in ionizing radiation intensity by absorbing material.

(5) $$MFP=\frac{1}{\mu }$$

The effective atomic number (Z*_eff_*) is a parameter that helps to express the interaction behaviour of compounds containing more than one type of element or composite materials, such as those formed in this study, with photons using a single atomic number. Just like elements with high atomic numbers, compounds or composite materials with high Z*_eff_* values are preferred in radiation shielding applications because they attenuate ionizing radiation more effectively. The Z*_eff_* value varies depending on the chemical composition, density, and photon energy of the material and plays an important role in evaluating radiation interaction mechanisms such as the photoelectric effect, Compton scattering, and pair formation [35]. The Z*_eff_* value is calculated according to Equation (6).

(6) $$Z_{eff}=\frac{\sigma_{a}}{\sigma_{e}}$$

where *σ_a_* represents the effective atomic cross-section, indicating the overall likelihood of interaction between incoming photons and the atoms in the substance, while *σ_e_* corresponds to the electronic cross-section, which reflects the contribution of each electron inside the atoms to the total interaction.

Effective electron density (N*_eff_*), a number representing the number of active electrons in a material (electron/g) that interact with radiation, is also considered an important parameter in the characterization of ionizing radiation shielding. The N*_eff_* value varies depending on the composition of the material and is functional in evaluating the scattering and absorption processes of photons within the material. High N*_eff_* values imply that the material interacts more effectively with radiation. The Neff value is determined by Equation (7):

(7) $$N_{eff}=\frac{N_{a}Z_{eff}}{\sum_{i}f_{i}A_{i}}$$

where $N_{a}\;$ is the Avogadro number (6.022 × 10^23^ mol^−1^), $f_{i}\;$ is the mole fraction of the i-th element, and $A_{i}\;$ represents the atomic weight of the i-th element (g∙mol^−1^) [36].

### 2.5. Radiation Shielding Experimental Setup

**Figure 5:**
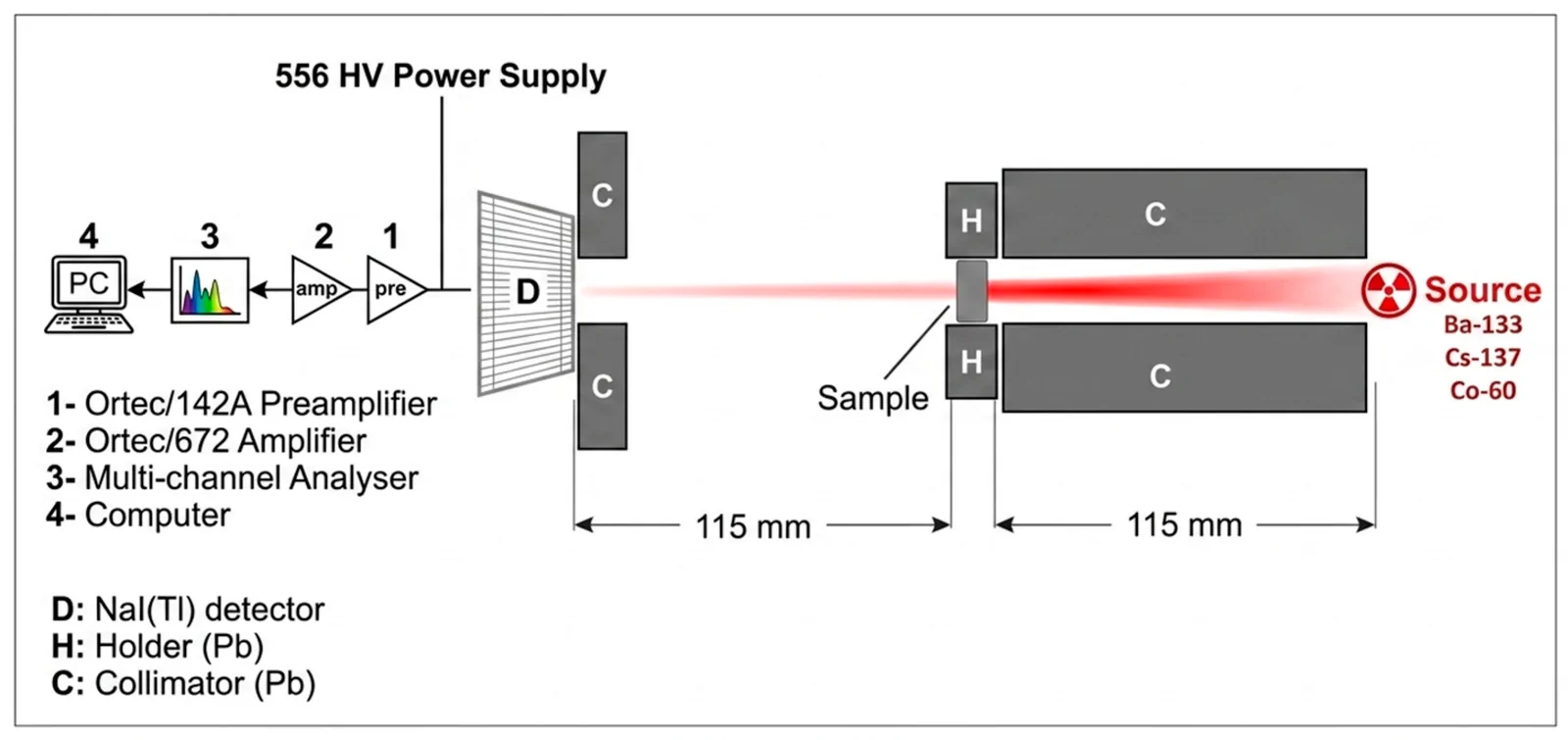
Setup diagram for gamma radiation shielding measurements.

Ionizing radiation attenuation experiments were conducted employing the narrow-beam transmission technique. As depicted in Figure 5, the samples were placed between the radioactive source and an ORTEC Model 905-4 NaI(Tl) detector (3 × 3 in.; AMETEK ORTEC, Oak Ridge, TN, USA), which was employed to record both attenuated and unattenuated gamma photonscoming from Ba-133, Cs-137, and Co-60 point sources, each with an activity of approximately 0.1 μCi. The distances from the sample to both the radioactive source and the detector were equal at 115 mm. The signals collected by the NaI(Tl) scintillation detector were transmitted to the photomultiplier tube (PMT) base (digiBASE, Ortec), with dimensions of 8.0 cm in length and 6.3 cm in diameter. The signals were subsequently amplified and processed through a multichannel analyzer interfaced with a computer, and the experimental spectra were analyzed using Maestro-32 software. To ensure reliability, each measurement was conducted three times (with a counting time of 20 min for each run), and the statistical uncertainty of the gamma-ray spectroscopy measurements was found to be about 2%.

### 2.6. Theoretical Calculations and Monte Carlo Simulations

Theoretical calculations and Monte Carlo simulations were performed to validate the experimentally obtained radiation shielding parameters and to investigate the interaction behavior of composites with gamma rays in more detail. In this context, the mass attenuation coefficients, as well as the effective atomic numbers, and effective electron densities, of the samples were calculated using the WinXCom (Version 1.0) program. Furthermore, the transport and attenuation behavior of photons at different energy levels within the material were determined by using the MCNP code.

Theoretical and simulation investigations were performed utilizing the chemical compositions listed in Table 2.

#### 2.6.1. Theoretical Determination of Radiation Shielding Parameters Using WinXCom

The theoretical mass attenuation coefficients (MACs) of the prepared samples were calculated using the WinXCom program, a Windows-based implementation of the XCOM photon cross-section database originally developed by Berger and Hubbell and later modernized by Gerward et al. [37]. As is known, the WinXCom program allows the calculation of MAC and total photon interaction cross-sections and partial cross-sections for individual processes including coherent and coherent scattering, photoelectric effect and pair formation for elements, compounds or mixtures using the mixing rule presented in Equation (8).

(8) $${\left(MAC\right)}_{m}=\sum_{i}^{n}w_{i}{\left(MAC\right)}_{i}$$

In Equation (8), (*MAC*)*_i_* and *w_i_* represent the mass attenuation coefficient along with the weight fraction of the i-th component, respectively.

#### 2.6.2. MCNP-Based Simulation of Radiation Attenuation Geometry Design

**Figure 6:**
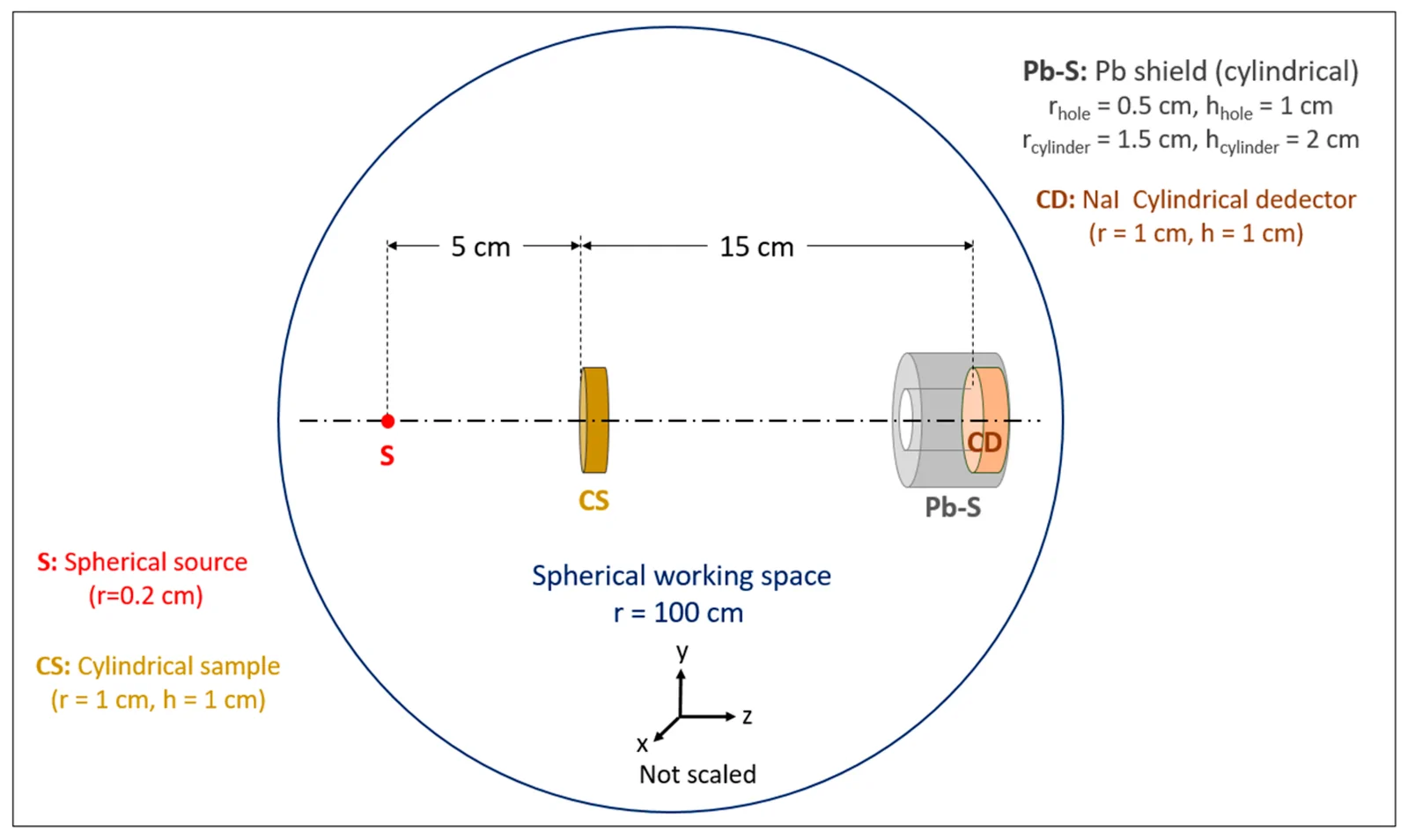
MCNP6.3 simulation geometry for determining the attenuation parameters. The dashed line represents the central photon-beam axis from the source through the sample to the detector.

MCNP6.3 is the most recent version of the Monte Carlo N-Particle (MCNP) transport code. The MCNP6.3 simulation geometry, shown in Figure 6, included the photon source, NaI(Tl) detector, and the samples, which were modeled as cylinders with a radius of 1 cm and height of 1 cm. The detector assembly was enclosed within a cylindrical lead shield, and the simulation environment was defined as a vacuum sphere with a 100 cm radius. The average photon flux within the geometry was evaluated using the f4 tally. Photon interactions were calculated using the ENDF/B-VI (Release 8) and MCPLIB04 data libraries, while the samples were defined via material cards based on their chemical compositions (Table 2). Simulations were performed at photon energies of 81, 356, and 662 keV, as well as 1173 and 1332 keV. Initially, the incident photon flux was determined without a sample, and subsequently, simulations were performed for both the pure UPR and the nanocomposites.

## 3. Results and Discussion

**Figure 7:**
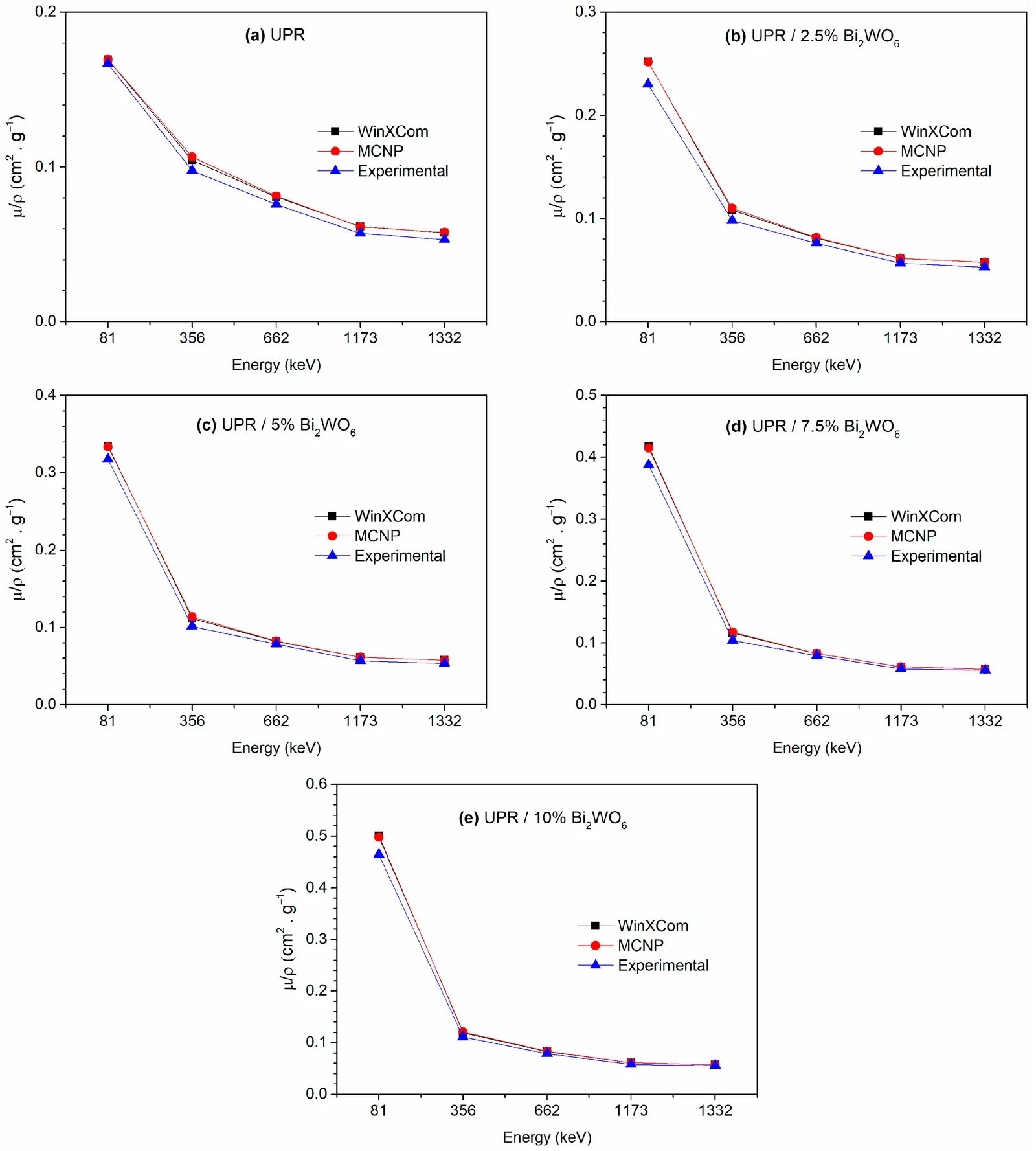
Comparison of the mass attenuation coefficient values of the samples.

Figure 7a–e shows the MAC values of pure UPR and UPR nanocomposites containing 2.5%, 5%, 7.5%, and 10% Bi_2_WO_6_ nanoparticles, respectively, at photon energies of 81, 356, 662, 1173, and 1332 keV, along with experimental measurements, WinXCom calculations, and MCNP6 simulation results. The mass attenuation coefficient of the pure UPR sample was first determined experimentally at different photon energies, and the obtained results were compared with WinXCom and MCNP calculations. The experimentally obtained MAC values for pure UPR were determined as 0.1666, 0.0976, 0.0757, 0.0571, and 0.0531 cm^2^g^−1^ at photon energies of 81, 356, 662, 1173, and 1332 keV, respectively. These values are in general good agreement with the MAC values of 0.1574, 0.1153, 0.0788, 0.0559, and 0.0459 cm^2^g^−1^ reported by Ghule et al. in the same energy range [29]. The differences are particularly small at energies of 662 and 1173 keV, while the limited deviations observed at lower energies are considered to be due to sample density, resin composition, fabrication method, and experimental uncertainties. The consistency of the experimental results with both theoretical calculations and literature data confirms the reliability of the established experimental system and the applied measurement methodology; therefore, it also supports the reliability of the radiation attenuation results obtained for Bi_2_WO_6_ nanoparticle-reinforced UPR nanocomposites.

As can also be seen from Figure 7, MAC values decrease continuously with increasing photon energy in all samples. The highest MAC values were obtained at 81 keV, while the lowest values were observed at 1332 keV. A particularly sharp decrease in MAC values occurs in the 81–356 keV energy range, while this decreasing trend slows down considerably at higher energies. This behavior is in great agreement with the energy-dependent changes in the interaction mechanisms of photons with matter. As is known, the probability of photon absorption is quite high due to the dominant photoelectric interaction between the incoming low-energy photons and the material. Conversely, as the energy of the incoming photon increases, the previously dominant photoelectric effect rapidly gives way to the dominant Compton scattering, and consequently, the MAC values decrease.

Examining Figure 7, the MAC values for pure UPR are the lowest at every photon energy among all samples. This is because UPR largely consists of elements with low atomic numbers. In contrast, it is observed that the MAC values systematically increase throughout the photon energy range as the amount of Bi_2_WO_6_ nanoparticles in the UPR matrix increases. This increase is particularly pronounced at 81 keV. The main reason for this is that the bismuth (Z = 83) and tungsten (Z = 74) atoms in the Bi_2_WO_6_ structure have high atomic numbers, significantly increasing the probability of photoelectric interaction. Thus, as the amount of Bi_2_WO_6_ in the composite increases, the probability of photons interacting with atoms of higher atomic number increases, and consequently, the attenuation performance improves.

In contrast, at energies of 662 keV and above, the effect of Bi_2_WO_6_ doping on MAC is more limited. This is because Compton scattering is the dominant interaction mechanism in this energy region. Since Compton scattering depends on electron density rather than atomic number, increasing the doping ratio does not provide as significant an improvement as at lower energies.

Among all the samples examined, the highest MAC values were obtained in the UPR nanocomposite containing 10% Bi_2_WO_6_, while pure UPR showed the lowest attenuation performance. These results demonstrate that Bi_2_WO_6_ nanoparticles are an effective radiation shielding additive within the UPR matrix, and that increasing the doping ratio significantly improves the gamma ray attenuation performance.

Another important finding from Figure 7 is the excellent agreement between the experimental measurements, WinXCom calculations, and MCNP6.3 simulation results. For all investigated nanocomposites, the three independent approaches yielded very similar MAC values over the entire photon energy range and for all Bi_2_WO_6_ concentrations. The largest deviations were observed at 81 keV, whereas the calculated and experimental values almost completely overlapped at photon energies of 356 keV and above. The maximum relative deviation between the experimental and MCNP results was 2.8%, while the deviation between the experimental and WinXCom results did not exceed 2.1%.

The slightly larger deviations observed at low photon energies can be attributed to unavoidable experimental uncertainties such as counting statistics, detector response, small variations in sample thickness and density, together with the ideal assumptions adopted in the WinXCom and MCNP6.3 calculations regarding material homogeneity and geometry. Nevertheless, the overall agreement demonstrates that these uncertainties have only a minor influence on the calculated shielding parameters and confirms the reliability of both the experimental measurements and the computational approaches employed in this study.

To provide a broader evaluation of the radiation shielding performance of the developed nanocomposite, the experimental mass attenuation coefficient (MAC) values obtained in this study were compared not only with previously reported UPR-based shielding composites but also with representative PMMA-based polymer composites containing Bi_2_O_3_ and WO_3_ high-Z fillers reported in the literature. The comparison was performed using experimental MAC values at 81 and 662 keV, representing the low- and medium-photon energy regions where the effect of Bi_2_WO_6_ incorporation is most significant. The comparative results are summarized in Table 3.

As summarized in Table 3, the developed UPR/10 wt.% Bi_2_WO_6_ nanocomposite exhibits excellent radiation attenuation performance compared with both UPR- and PMMA-based shielding composites reported in the literature. At 81 keV, the experimental MAC value of 0.4638 cm^2^ g^−1^ is higher than those reported for UPR composites reinforced with Bi_2_O_3_ and Bi_2_(WO_4_)_3_, as well as the reported PMMA/10 wt.% Bi_2_O_3_ composite. Similarly, at 662 keV, the developed nanocomposite exhibits an MAC value (0.0757 cm^2^ g^−1^) that is comparable to the reported PMMA/10 wt.% WO_3_ composite while requiring only 10 wt.% filler loading. These results demonstrate that Bi_2_WO_6_ nanoparticles are highly effective shielding fillers for polymer composites and confirm that the developed UPR/Bi_2_WO_6_ nanocomposite provides competitive radiation shielding performance despite its relatively low filler content.

**Figure 8:**
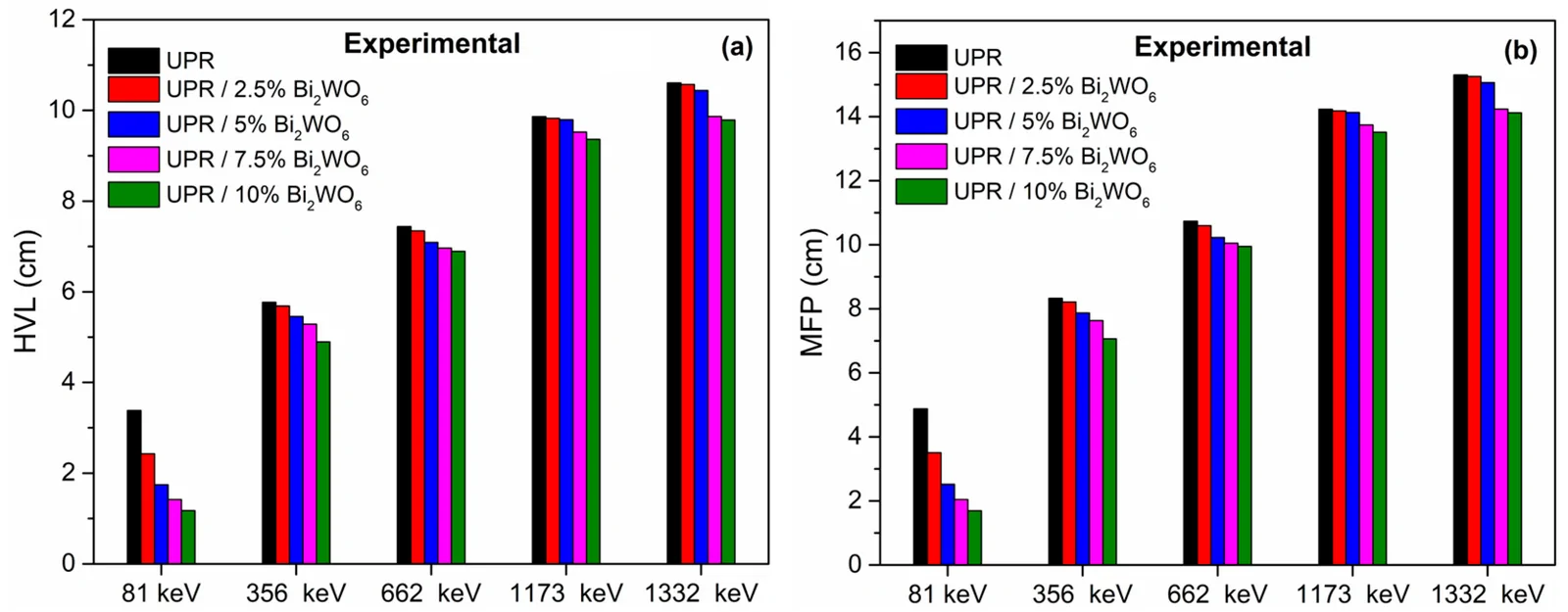
Comparison of experimentally determined (**a**) HVL and (**b**) MFP values of the samples at photon energies of 81, 356, 662, 1173 and 1332 keV.

Figure 8 shows the experimentally determined half-value layer and mean free path values of the pure UPR and the UPR based nanocomposites containing different ratios of Bi_2_WO_6_ nanoparticles (2.5%, 5%, 7.5%, and 10%, by weight) at photon energies of 81, 356, 662, 1173, and 1332 keV. As seen in Figure 8, both HVL and MFP values increase with increasing photon energy in all samples. This is due to the fact that as the energy increases, the photons attenuate over longer distances within the material, and therefore, larger material thicknesses are needed to achieve the same attenuation level. However, it is observed that the HVL and MFP values decrease regularly at all photon energies with increasing Bi_2_WO_6_ doping ratio. The lowest HVL and MFP values were obtained in the UPR nanocomposite with 10% Bi_2_WO_6_ doping. This result demonstrates that Bi_2_WO_6_ nanoparticles enhance the photon attenuation capacity of the UPR matrix, and thinner material thicknesses are sufficient to achieve the same radiation attenuation performance. The doping ratio-dependent reduction is particularly pronounced at 81 keV, with a gradual decrease in the difference between samples at higher energies. This trend is consistent with the MAC results, confirming that Bi_2_WO_6_ doping significantly improves radiation shielding performance, especially against low-energy photons.

**Figure 9:**
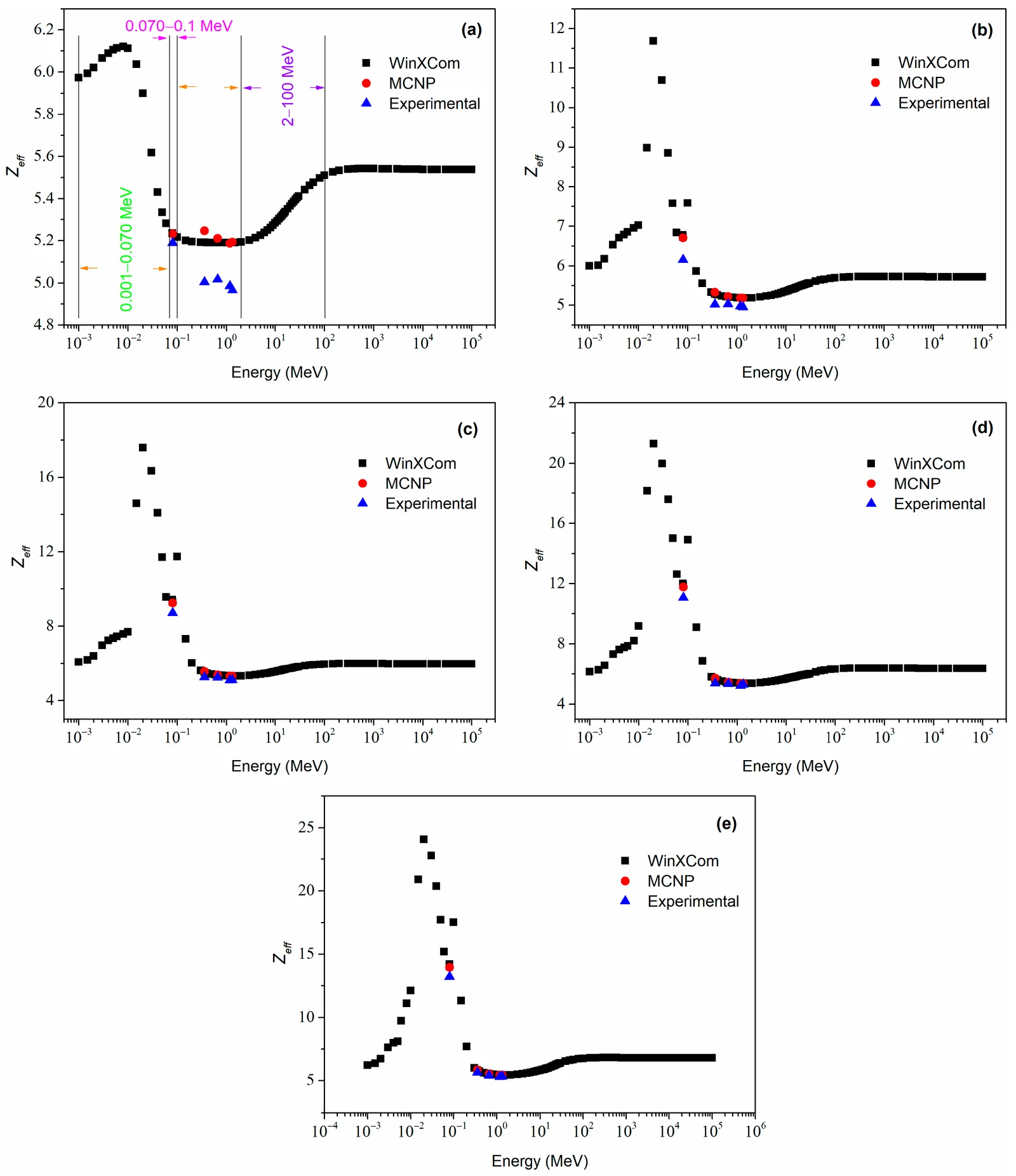
Comparison of the effective atomic number values of the (**a**) Neat UPR, (**b**) UPR/2.5% Bi_2_WO_6_, (**c**) UPR/5% Bi_2_WO_6_, (**d**) UPR/7.5% Bi_2_WO_6_, and (**e**) UPR/10% Bi_2_WO_6_ obtained from WinXCom calculations (1 keV–100 GeV), MCNP6.3 simulations, and experimental measurements.

**Figure 10:**
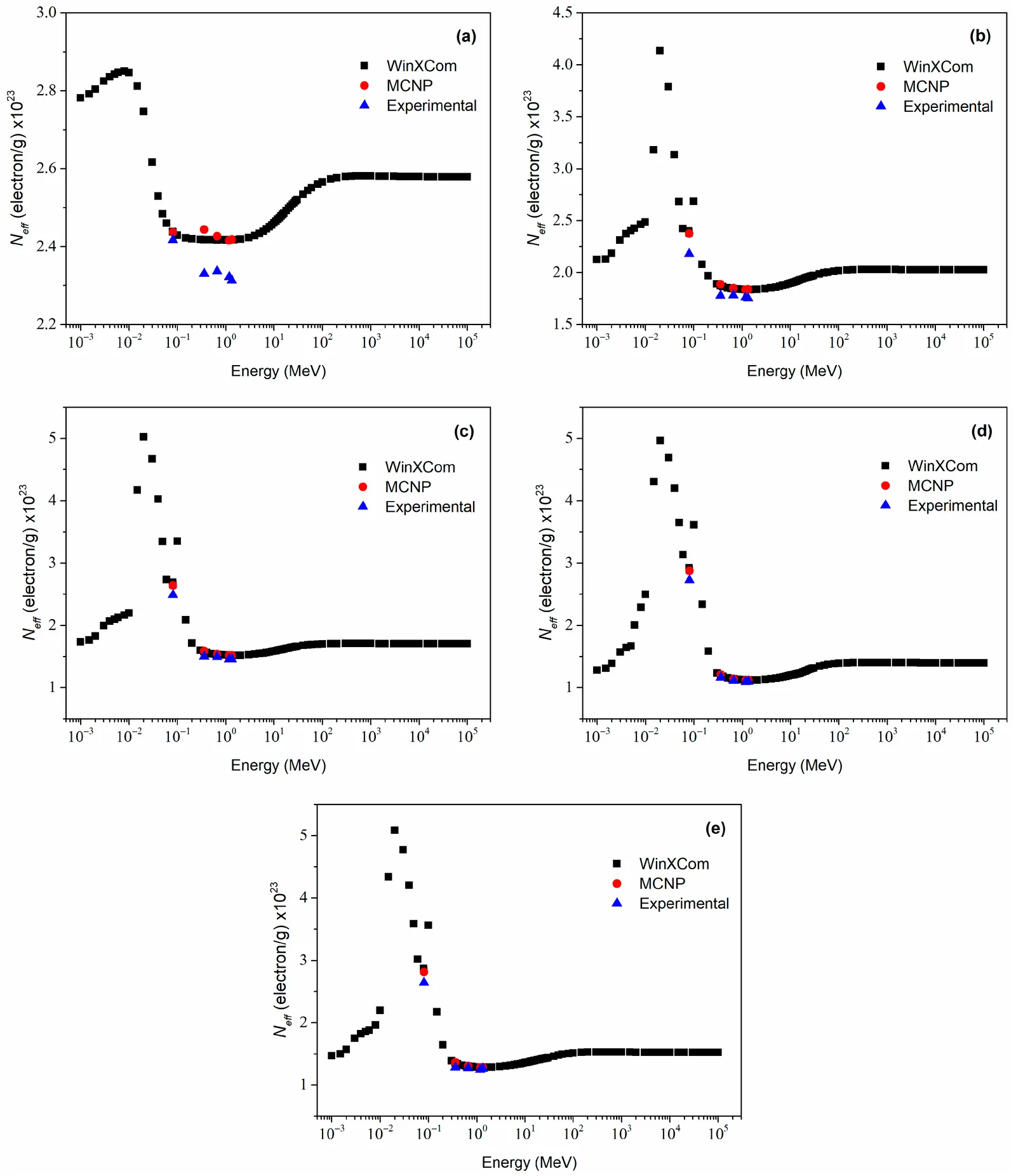
Comparison of the effective electron density values of the (**a**) Neat UPR, (**b**) UPR/2.5% Bi_2_WO_6_, (**c**) UPR/5% Bi_2_WO_6_, (**d**) UPR/7.5% Bi_2_WO_6_, and (**e**) UPR/10% Bi_2_WO_6_ obtained from WinXCom calculations (1 keV–100 GeV), MCNP6.3 simulations, and experimental measurements.

Further analysis was conducted on Z*_eff_* and N*_eff_*, which are known to be interdependent and critical in evaluating gamma-ray attenuation behaviour. Figure 9 and Figure 10 depict the variations in the effective atomic number and effective electron density, respectively, as functions of photon energy for the samples.

The energy-dependent change trends obtained for both parameters appear to be quite similar. This is an expected result, as both parameters are similarly affected by changes in photon–matter interaction mechanisms since N*_eff_* values are calculated directly from Z*_eff_* values.

Examining Figure 9 and Figure 10, it is observed that the experimental values deviate slightly from the WinXCom curves in pure UPR and low Bi_2_WO_6_ dopant ratios (2.5% and 5%), while the experimental, MCNP6.3, and WinXCom results converge significantly when the dopant ratio reaches 7.5% and especially 10%. This can be explained by the Bi_2_WO_6_ nanoparticles becoming more dominant in the matrix with increasing dopant ratio and the effective atomic number of the composite better approaching the homogeneous composition assumed in theoretical calculations. Furthermore, since the probability of photons interacting with high atomic number Bi and W atoms increases at high dopant ratios, the experimental results more successfully represent the theoretical models based on the ideal mixture assumption.

As shown in Figure 9 and Figure 10, the energy-dependent variations in the effective atomic number (Z*_eff_*) and effective electron density (N*_eff_*) are determined by the dominant photon–matter interaction mechanism. In the low energy region (<100 keV), the photoelectric effect is dominant, and the photoelectric cross-section approximately follows the relation *σ* ∝ Z^4–5^/E^3–3.5^. Therefore, in Bi_2_WO_6_-doped composites containing elements with high atomic numbers Bi and W, the Z*_eff_* and N*_eff_* values are significantly increased compared to the pure UPR. The abrupt changes observed in the energy range of approximately 70–100 keV originate from the K-absorption edges of tungsten (69.5 keV) and bismuth (90.5 keV) [40]. In the mid-energy region (≈100 keV–2 MeV), the dominant interaction is Compton scattering. The Compton cross-section is electron density-dependent and has a very weak dependence on atomic number (*σ* ∝ *Z*). Therefore, Z*_eff_* and N*_eff_* values approach each other and reach their minimum levels in all samples. At higher energies (>2 MeV), pair production becomes increasingly dominant, and the cross-section changes to approximately *σ* ∝ *Z*^2^ [41]. As a result, the effect of high atomic number dopants increases again, Z*_eff_* and N*_eff_* values rise again, and plateau behavior is exhibited in the high-energy region. Furthermore, it is observed that with increasing Bi_2_WO_6_ dopant ratio, experimental and MCNP results converge more closely to WinXCom calculations. This can be explained by the fact that the high-Z dopant phase strengthens the effective atomic composition in the composite and increases the agreement between theoretical calculations and experimental results.

## 4. Conclusions

In this study, Bi_2_WO_6_ nanoparticles synthesized by the hydrothermal method were added to unsaturated polyester resin at different weight ratios to produce UPR-based nanocomposites, and their radiation shielding performance against ionizing gamma rays was investigated experimentally and theoretically. XRD, FTIR, and FESEM analyses determined the structure and morphology of the synthesized Bi_2_WO_6_ nanoparticles in accordance with the scientific literature.

Experimental results revealed that the mass attenuation coefficients increased hierarchically with increasing Bi_2_WO_6_ doping ratio, while the HVL, TVL, and MFP values similarly decreased gradually. This improvement was particularly pronounced at low photon energies and is due to the high atomic numbers of Bi and W atoms in the energy region where the photoelectric effect is dominant. The UPR nanocomposite with 10% Bi_2_WO_6_ doping showed the highest radiation attenuation performance among all composites studied, with an MAC value of 0.4638 cm^2^∙g^−1^ at 81 keV. The experimental results showed high agreement with WinXCom theoretical calculations and MCNP6.3 Monte Carlo simulations, with the highest relative deviation between experimental and theoretical results remaining below 2.8%. Z*_eff_* and N*_eff_* analyses also confirmed that Bi_2_WO_6_ doping significantly increased the probability of photon–matter interaction in the low-energy region; the characteristic changes observed in the WinXCom curves were determined to originate from the K-absorption edges of the W (69.5 keV) and Bi (90.5 keV) elements. Comparisons with similar UPR composites doped with Bi_2_O_3_, Bi_2_(WO_4_)_3_, WO_3_, and SnO_2_, as reported in the scientific literature, have shown that the radiation shielding performance obtained with only 10% Bi_2_WO_6_ doping is higher than or comparable to many similar systems. This indicates that Bi_2_WO_6_ nanoparticles are a highly effective radiation shielding additive for UPR matrices.

In conclusion, Bi_2_WO_6_-doped UPR nanocomposites are considered a significant candidate among next-generation lead-free radiation shielding materials that can be used in medical radiation protection, nuclear technology, industrial radiography, and other ionizing radiation applications, thanks to their low- and medium-energy gamma ray attenuation performance, remarkable shielding at low doping rates, lead-free nature, low weight, and ease of processing.

## Figures and Tables

**Table 1:** Mass density and % relative heaviness of the composites, along with their components.

Samples	Mass Density (g/cm^−3^)	RH (%)
Pb	11.3400	100
UPR	1.2307	10.8527
Bi_2_WO_6_	9.5200	83.9506
UPR/2.5% Bi_2_WO_6_	1.2417	10.9497
UPR/5% Bi_2_WO_6_	1.2503	11.0256
UPR/7.5% Bi_2_WO_6_	1.2611	11.1208
UPR/10% Bi_2_WO_6_	1.2737	11.2319

**Table 2:** Elemental contents of the Neat UPR, Bi_2_WO_6_ and the UPR/Bi_2_WO_6_ nanocomposites.

Sample	Atoms						Total
	H	C	O	Co	W	Bi	
UPR	5.692 × 10^−2^	7.324 × 10^−1^	2.105 × 10^−1^	1.800 × 10^−4^	0	0	1
Bi_2_WO_6_	0	0	1.375 × 10^−1^	0	2.635 × 10^−1^	5.990 × 10^−1^	1
UPR/2.5% Bi_2_WO_6_	5.550 × 10^−2^	7.141 × 10^−1^	2.087 × 10^−1^	1.755 × 10^−4^	6.590 × 10^−3^	1.498 × 10^−2^	1
UPR/5% Bi_2_WO_6_	5.407 × 10^−2^	6.958 × 10^−1^	2.069 × 10^−1^	1.710 × 10^−4^	1.318 × 10^−2^	2.995 × 10^−2^	1
UPR/7.5% Bi_2_WO_6_	5.265 × 10^−2^	6.775 × 10^−1^	2.050 × 10^−1^	1.665 × 10^−4^	1.976 × 10^−2^	4.493 × 10^−2^	1
UPR/10% Bi_2_WO_6_	5.123 × 10^−2^	6.592 × 10^−1^	2.032 × 10^−1^	1.620 × 10^−4^	2.635 × 10^−2^	5.990 × 10^−2^	1

**Table 3:** Comparison of the experimental MAC value of the UPR/10% Bi_2_WO_6_ nanocomposite developed in this study at low and mid-photon energies with different UPR-based composites reported in the scientific literature.

Sample	MAC Value (cm^2^g^−1^)	Reference
UPR/10% Bi_2_O_3_	0.3552 at 81 keV	[29]
UPR/40%WO_3_	0.094 at 662 keV	[38]
UPR/5%Bi_2_(WO_4_)_3_	0.3485 at 81 keV	[31]
UPR/50%SnO_2_	0.3523 at 122 keV	[30]
PMMA/10%Bi_2_O_3_	<0.400 at 80 keV	[39]
PMMA/10%WO_3_	0.0762 at 662 keV	[17]
UPR/10%Bi_2_WO_6_	0.4638 at 81 keV 0.0757 at 662 keV	Present Study

## Data Availability

The original contributions presented in this study are included in the article. Further inquiries can be directed to the corresponding author.

## Abbreviations

The following abbreviations are used in this manuscript:

Bi_2_O_3_	Bismuth(III) Oxide
Bi_2_WO_6_	Bismuth Tungstate
FESEM	Field Emission Scanning Electron Microscopy
HVL	Half-Value Layer
MAC	Mass Attenuation Coefficient
MCNP	Monte Carlo N-Particle Transport Code
MFP	Mean Free Path
Neff	Effective Electron Density
PMMA	Poly(methyl methacrylate)
PTFE	Polytetrafluoroethylene
PVC	Poly(vinyl chloride)
PVP	Polyvinylpyrrolidone
PEG	Polyethylene Glycol
TVL	Tenth-Value Layer
UPR	Unsaturated Polyester Resin
XRD	X-ray Diffraction
Zeff	Effective Atomic Number
